# Hematopoietic 12/15-lipoxygenase activity negatively contributes to fungal-associated allergic asthma

**DOI:** 10.1152/ajplung.00090.2023

**Published:** 2023-05-30

**Authors:** Mgayya Makullah, Diandra A. Ellis, MaryJane Jones, Chad Steele

**Affiliations:** Department of Microbiology and Immunology, Tulane University, New Orleans, Louisiana, United States

**Keywords:** allergy, asthma, fungi, immunopathogenesis, inflammation

## Abstract

Asthma is one of the most common noncommunicable diseases in the world. Approximately 30% of severe cases are associated with fungal sensitization, often associated with allergy to the opportunistic mold *Aspergillus fumigatus*. Leukotrienes, immunopathogenic mediators derived from the metabolism of arachidonic acid (AA) by 5-lipoxygenase (5-LOX), are often elevated in severe asthma. As such, these mediators are Food and Drug Administration-approved therapeutic targets of the antiasthmatic drugs Zileuton/Zyflo and Singulair/Montelukast. A second enzyme involved in AA metabolism is 12/15-lipoxygenase (12/15-LOX; *Alox15*). Here, C57BL/6 wild-type (WT) mice subjected to experimental fungal asthma had increased expression of *Alox15* mRNA and increased levels of 12-HETE, a product of 12/15-LOX activity, in the lung when compared with naïve and vehicle-treated mice. Mice deficient in 12/15-LOX (*Alox15*^−/−^) demonstrated better lung function, as measured by airway hyperresponsiveness (AHR), during fungal asthma. Histological assessment revealed reduced inflammation in the lungs of *Alox15*^−/−^ mice compared with WT mice, which was corroborated by flow cytometric analysis of multiple myeloid (eosinophils and neutrophils) and lymphoid (CD4+ T and γδ T) cell populations. This was further supported by decreased levels of specific chemokines that promote the recruitment of these cells. Likewise, type 1 and 2, but not type 17 cytokines, were significantly lower in the lungs of *Alox15*^−/−^ mice. Bone marrow chimera studies revealed that the presence of 12/15-LOX in hematopoietic cells contributed to AHR during fungal asthma. Taken together, our data support the hypothesis that hematopoietic-associated 12/15-LOX contributes to type 1 and 2 responses and exacerbation of allergic fungal asthma.

**NEW & NOTEWORTHY** Humans with asthma sensitized to fungi often have more severe asthma than those who are not sensitized to fungi. Products of arachidonic acid generated via 5-lipoxygenase are often elevated in severe asthma and are successful FDA-approved drug targets. Less understood is the role of products generated via 12/15-lipoxygenase. We demonstrate that 12/15-lipoxygenase expression in hematopoietic cells contributes to type 1 and 2 responses and impaired lung function during allergic fungal asthma.

## Graphical abstract

**Figure:**
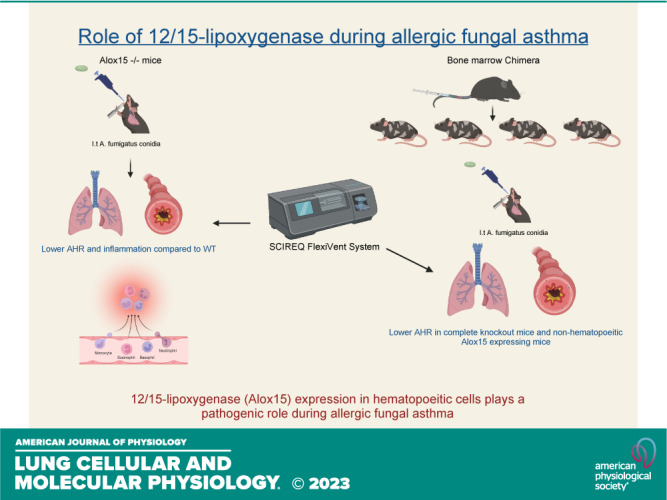

## INTRODUCTION

Asthma is one of the most common chronic respiratory conditions with significant public health consequences and occurs in patients of all ages. In the United States, over 25 million people are diagnosed with asthma, and over 300 million worldwide (1). Asthma is an inflammatory disease characterized by airway obstruction and hyperresponsiveness that results in chest tightness, dyspnea, coughing, and wheezing (1–3). About 10% of asthma cases are termed severe due to significant physiological and functional impacts on these patients. Patients experience frequent exacerbations, require higher doses of corticosteroids/long-acting bronchodilator therapy, and have higher mortality and morbidity (2, 3). Of these severe cases, ∼30% are sensitized to filamentous fungi, most commonly *Aspergillus fumigatus* (3). This represents a specific asthma phenotype known as severe asthma with fungal sensitization (SAFS) (2, 3).

The etiology of asthma is not entirely understood. However, studies have been able to identify key players involved in the development of asthma. Several bioactive lipid mediators derived from arachidonic acid (AA) metabolism play an important role in mediating inflammation and asthma (4–6). AA metabolism is catalyzed by lipoxygenases (LOX), cyclooxygenases (COX), and cytochrome p450 (CYP) enzymes (7). Regarding LOX enzymes, research shows that 5-LOX activity has a significant role in promoting inflammation in asthma. 5-LOX activity leads to the production of leukotriene B4 (LTB4) and cysteinyl leukotrienes (CysLT) such as LTC4, LTD4, and LTE4 (4–6, 8–10). LTB4 is a potent chemoattractant involved in the recruitment and activation of antigen-presenting cells and T cells (6, 10). CysLTs are the primary metabolites of 5-LOX activity in many cell types and are considered important mediators of asthma pathogenesis. They are potent bronchoconstrictors involved with eosinophil recruitment, mucus secretion, and airway remodeling. In addition, cysLTs are directly linked with inducing Th2 cell differentiation and cytokine production (6, 10). Consequently, investigators have sought to find therapeutic interventions to target the 5-LOX pathway. Indeed, an inhibitor of 5-LOX, Zileuton, was Food and Drug Administration (FDA)-approved for prophylactic and chronic treatment of asthma in 1997 (11). Later, cysLT receptor antagonists, Montelukast and Zafirlukast, were developed as alternative therapies targeting the 5-LOX pathway (9). Identifying the significant role 5-LOX plays in mediating asthma poses an interesting question about the role and therapeutic potential of other LOX enzymes, particularly 12/15-LOX, in asthma.

12/15-LOX enzyme activity generates proinflammatory mediators such as hydroxyeicosatetraenoic acids (HETE) and hepoxilins, as well as anti-inflammatory compounds including lipoxins, resolvins, and protectins (6, 12, 13). Several reports indicate the double-edged nature of 12/15-LOX. In one study, deletion of 12/15-LOX exacerbated inflammation in arthritic mice caused by low levels of lipoxin A4 and protectin (13, 14). In contrast, a colitis model observed an opposite effect where 12/15-LOX deletion resulted in a less severe inflammatory phenotype (13). We have previously shown that 12/15-LOX plays an essential role in lung proinflammatory responses to *A. fumigatus* during invasive aspergillosis (15). Mice deficient in 12/15-LOX have higher lung fungal burden and low survival rate as a result of impaired inflammatory response in the early stages of infections (15). In this study, we sought to elucidate the role of 12/15-LOX in allergic fungal asthma and its impact on lung function.

## MATERIALS AND METHODS

### Mice

Male and female, age-matched C57BL/6 mice, 6 to 8 wk of age, were obtained from The Jackson Laboratory (Bangor, ME). Male and female, age-matched *Alox15*^−/−^ mice were obtained from The Jackson Laboratory (B6.129S2-*Alox15^tm1Fun^*/J, Stock No: 002778) and bred at Tulane University. All animals were housed in a specific pathogen-free, Association for Assessment and Accreditation of Laboratory Animal Care-certified facility and handled according to Public Health Service Office of Laboratory Animal Welfare policies after review and approval by the Tulane Institutional Animal Care and Use Committee.

### Preparation of *A. fumigatus* and Chronic In Vivo Challenge

*A. fumigatus* isolate 13073 (ATCC, Manassas, VA) was maintained on potato dextrose agar for 5–7 days at 37°C. Conidia were harvested by washing the culture flask with 50 mL of sterile phosphate-buffered saline supplemented with 0.1% Tween 20. The conidia were then passed through a sterile 40-μm nylon membrane to remove hyphal fragments and enumerated on a hemacytometer. The repeated *A*. *fumigatus-*associated allergic airway inflammation model was performed as we have extensively reported (16–18). Briefly, mice were lightly anesthetized with isoflurane and administered 1 × 10^7^ live *A. fumigatus* conidia in a volume of 50 μL of PBS intratracheally (it). After resting for 7 days, mice were challenged intratracheally with 1 × 10^6^ live *A. fumigatus* conidia in 50 μL of PBS daily for five consecutive days (*days 7, 8, 9, 10*, and *11*), allowed to rest for two consecutive days (*days 12* and *13*), and then challenged intratracheally with 1 × 10^6^ live *A. fumigatus* conidia in 50 μL of PBS daily for three consecutive days (*days 14, 15,* and *16*). Twenty-four hours after the last *A. fumigatus* challenge (*day 17*), mice were euthanized for assessment of various outcome measures. Mice in the vehicle group were administered PBS, i.e., at identical time points as mice in the asthmatic group.

### *Alox15* mRNA and 12-HETE Analysis

For real-time PCR analysis of *Alox15* gene expression, left lungs were collected into Trizol Reagent (Life Technologies, Carlsbad, CA), homogenized, and RNA was isolated according to the manufacturer’s protocol. mRNA levels were then measured via Taqman assay for *Alox15* and normalized to *Hprt* (Applied Biosystems, Waltham, MA). For 12-HETE analysis, lungs were collected, homogenized, and clarified by centrifugation. 12-HETE levels were quantified using 12(S)-HETE ELISA kit per the manufacturer’s instructions (Cat. AB133034, Abcam, Waltham, MA).

### Whole Lung Cytokine and Chemokine Analysis

Following euthanization, the right lung was homogenized in PBS supplemented with Complete Mini protease inhibitor tablets (Roche Diagnostics), clarified by centrifugation (12,000 *g* for 10 min at 4°C), and stored at −80°C. Clarified lung homogenate supernatants were analyzed for the protein levels of 32 cytokines and chemokines using the Luminex-based Milliplex multiplex suspension cytokine array (MilliporeSigma), according to the manufacturer’s instructions. The data were analyzed using Bio-Plex Manager software (Bio-Rad). IL-33, CCL17, and CCL22 levels were quantified by ELISA (R&D Systems).

### Lung Cell Flow Cytometry

Lung cells were isolated via bronchoalveolar lavage (BAL) as previously described (19). Cells were washed, and Fc receptors were blocked with Mouse BD Fc Block (BD Biosciences) at 4°C for 20 min. Thereafter, cells were stained with a single-color LIVE/DEAD Fixable Dead Cell Stain (Invitrogen), followed by labeling with specific immune cell surface markers. The following staining parameters were employed: eosinophils as CD45^+ ^CD11b^+^Siglec F^ + ^Ly-6G^–^ using anti-CD45 (BioLegend, Cat. No. 103115, clone 30-F11), anti-CD11b (BioLegend, Cat. No. 101237, clone M1/70), anti–Siglec F (Miltenyi Biotec, Cat. No. 130-123-816, clone ES22-10D8), and anti–Ly-6G (BioLegend, Cat. No. 127621, clone 1A8); neutrophils as CD45^ + ^CD11b^+^Ly6G^ + ^Siglec F^–^ using anti-CD45, anti CD11b, anti–Ly-6G, and anti–Siglec F; inflammatory monocytes as CD45 + CD11b+CD11c-CCR2 + F4/80-Ly6C+ using anti-CD45, anti-CD11b, anti-CD11c (BioLegend, Cat. No. 117308, clone N418), anti-CCR2 (BioLegend, Cat. No. 150605, clone SA203G11), anti-F4/80 (BioLegend, Cat. No. 123146, clone BM8), anti-Ly6C (BioLegend, Cat. No. 128012, clone HK1.4); dendritic cells (DCs) as CD45 + CD11c+Ly6C + MHCII+ using anti-CD45, anti-CD11c, anti–Ly6C, and anti–MHC II (BioLegend, Cat. No. 107630, clone M5/114.15.2); CD4 T cells as CD45 + CD3+CD4 + TCRb+ using anti-CD45, anti-CD3 (BioLegend, Cat. No. 100216, clone 17A2), anti–CD4 (BioLegend, Cat. No. 100422, clone GK1.5), and anti–TCRb (BioLegend, Cat. No. 109240, clone H57-597); γδ T cells as CD45 + CD3+TCRgd+ using anti-CD45, anti-CD3, and anti-TCRgd (BioLegend, Cat. No. 118117, clone GL3). Samples were acquired using a 4-laser, 20-parameter analytic BD LSRFortessa, and data were analyzed using FlowJo software (Tree Star Inc., Ashland, OR). Unstained lung leukocytes served as a control for background fluorescence and gating. Appropriately stained UltraComp eBeads (Thermo Fisher Scientific, Waltham, MA) served as single-color controls.

### Pulmonary Function Assessment

Individual anesthetized *A*. *fumigatus*-exposed mice were intubated, and each animal was attached to a computer-controlled volume ventilator (flexiVent; SCIREQ). Regular breathing was set at 150 breaths per minute, with volume and pressure controlled by the flexiVent system based on individual animal weights. Positive end-expiratory pressure was set to 2 cmH_2_O and measured during each breath stroke. The single-frequency forced oscillation technique was used to measure total/dynamic lung resistance (Rrs). The low-frequency/broadband forced oscillation technique was employed to measure Newtonian resistance (Rn; also known as airway hyperreactivity, AHR). All measurements were collected at baseline and after a linear dose response with methacholine challenge (10–50 mg/mL), as previously described (16–18, 20). Lung function was also assessed in naive WT and mutant mice, which confirmed no baseline anomalies and no differences between groups (data not shown).

### Bone Marrow Chimeras

WT and *Alox15*^−/−^ reciprocal chimeric mice were generated by irradiating the indicated recipient mice with 950 Rads from a gamma source (Cesium-137) delivered in one dose (19, 21). Following irradiation, mice were intravenously injected with 5 million total bone marrow cells (100% from either WT or *Alox15*^−/−^ mice) and were allowed to reconstitute for 8 wk before being subjected to experimental fungal asthma.

### Histology

Lungs were inflated and fixed in 4% formalin. The fixed lungs were paraffin-embedded and then processed and stained by GNO Histology Consultants (New Orleans, LA). Imaging was performed using a Swift Optical Instruments M10T-P Trinocular LED Microscope equipped with a Motic Moticam 5 + 5-megapixel digital camera

### Statistics

Data were analyzed using GraphPad Prism Version 9.5 statistical software (GraphPad Software). Comparisons between groups, when data were normally distributed, were made with the two-tailed unpaired Student’s *t* test or two-way-ANOVA. Significance was accepted at a value of *P* < 0.05.

## RESULTS

### 12/15-Lipoxygenase Is Induced in Mice Subjected to Allergic Fungal Asthma

**Figure 1.**
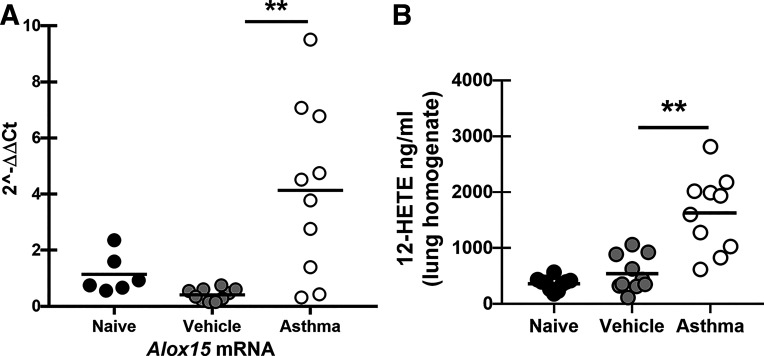
12/15-lipoxygenase is induced in mice subjected to allergic fungal asthma. *A*: C57BL/6 wild-type (WT) mice were subjected to the experimental allergic fungal asthma model as described in materials and methods. Mice in the vehicle group were administered PBS at identical time points as mice in the asthmatic group that received *Aspergillus fumigatus*. Twenty-four hours after the last challenge, the left lungs were collected and *Alox15* gene expression was quantified by real-time PCR and normalized to HPRT. Data are expressed as 2^−ΔΔCt^. The figure illustrates cumulative data from two independent studies (*n* = 3 mice per group per study for naïve mice and *n* = 5 mice per group per study for vehicle treated vs. asthmatic mice). Each dot presents a single mouse. The middle line represents the mean. **P < 0.01 (two-tailed Student’s *t* test). *B*: C57BL/6 wild-type (WT) mice were subjected to fungal asthma as in *A*. Twenty-four hours after the last challenge, the right lungs were homogenized and clarified by centrifugation and the levels of 12-HETE were quantified via ELISA. The figure illustrates cumulative data from two independent studies (*n* = 5 mice per group per study). Each dot presents a single mouse. The middle line represents the mean. **P < 0.01 (two-tailed Student’s *t* test). HETE, hydroxyeicosatetraenoic acid.

We have recently reported that normal C57BL/6 mice acutely exposed to *A. fumigatus* had increased lung levels of 12-hydroxyeicosatetraenoic acid (12-HETE), a major product of 12/15-lipoxygenase (12/15-LOX) activity, in the lung when compared with naïve mice (15). To confirm that 12/15-LOX was likewise induced during allergic fungal asthma, real-time PCR was used to quantify changes in *Alox15* mRNA expression between naïve, vehicle-treated, and mice subjected to allergic fungal asthma. Results showed an average fourfold increase in *Alox15* mRNA levels in asthmatic mice (Fig. 1*A*). This paralleled higher levels of 12-HETE in the lungs of asthmatic mice compared with vehicle-treated mice (Fig. 1*B*). Thus, *Alox15*/12/15-LOX is induced and active during allergic fungal asthma.

### 12/15-Lipoxygenase Contributes to Worse Airway Function during Allergic Fungal Asthma

**Figure 2.**
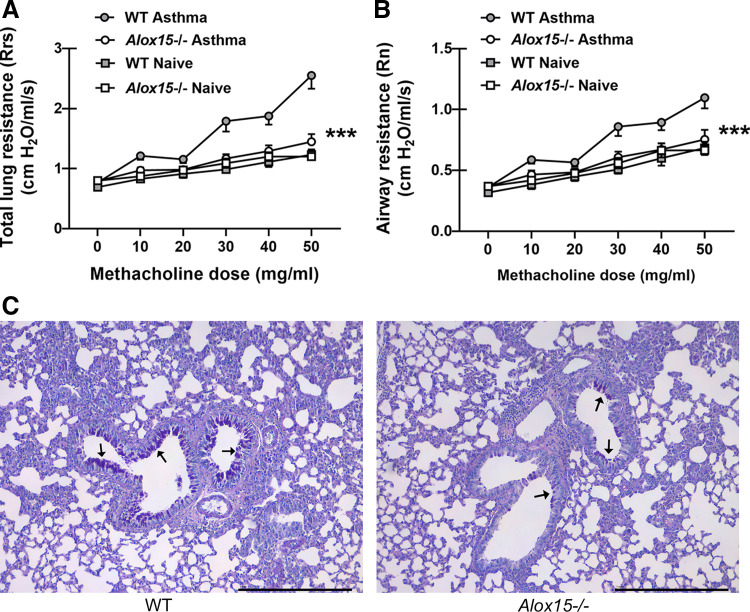
12/15-lipoxygenase (LOX) contributes to worse airway function during allergic fungal asthma. *A* and *B*: C57BL/6 wild-type (WT) and 12/15-LOX-deficient (*Alox15*^−/−^) mice were subjected to the experimental allergic fungal asthma model as described in materials and methods. Twenty-four hours after the last challenge, total lung resistance (*A*) and airway (Newtonian) resistance (*B*) were analyzed via mechanical ventilation using the flexiVent pulmonary function system. Naïve mice served as controls for baseline lung function. The figures illustrate cumulative data from two independent studies (*n* = 5 mice per group per study). Data are expressed as means ± SE. ***P < 0.001 when comparing asthmatic WT and *Alox15*^−/−^ mice (two-way ANOVA). *C*: representative Periodic acid Schiff-stained lung sections from asthmatic WT (*left*) and *Alox15*^−/−^ (*right*) mice. Original magnification ×10. Bar = 1 mm. Black arrows represent areas of mucus production.

Although not extensively investigated, studies suggest that 12/15-LOX may function in either promoting or protecting against allergic/asthmatic features (22–25). Importantly, however, the effects of 12/15-LOX activity on lung function during allergic asthma with a relevant aeroallergen, such as house dust mite or mold, has not been determined. Results here demonstrate that mice deficient in 12/15-LOX (*Alox15*^−/−^) had significantly lower total lung dynamic resistance (Fig. 2*A*) and airway hyperresponsiveness (AHR) (Fig. 2*B*) during *A. fumigatus*-associated allergic fungal asthma. The impact of 12/15-LOX was quite striking when comparing lung function between asthmatic and naïve mice, in that *Alox15*^−/−^ mice had little change in lung function after being subjected to experimental allergic fungal asthma. Of note, there was no difference in fungal burden between asthmatic WT and *Alox15*^−/−^ mice (24 h after the last fungal challenge on *day 16*, *A. fumigatus* lung burden was 4.58 × 10^3^ ± 5.7 × 10^2^ vs. 4.08 × 10^3^ ± 8.4 × 10^2^ in WT vs. *Alox15*^−/−^ mice, respectively; *P* = 0.653). Examination of mucus production via Periodic acid-Schiff (PAS) staining lung sections revealed increased goblet cell hyperplasia and airway mucus in WT mice (Fig. 2*C*, *left*) compared with *Alox15*^−/−^ mice (Fig. 2*C*, *right*). Thus, 12/15-LOX is a pathogenic mediator of disease severity during allergic fungal asthma.

### 12/15-Lipoxygenase Promotes Increased Lung Cellularity during Allergic Fungal Asthma

**Figure 3.**
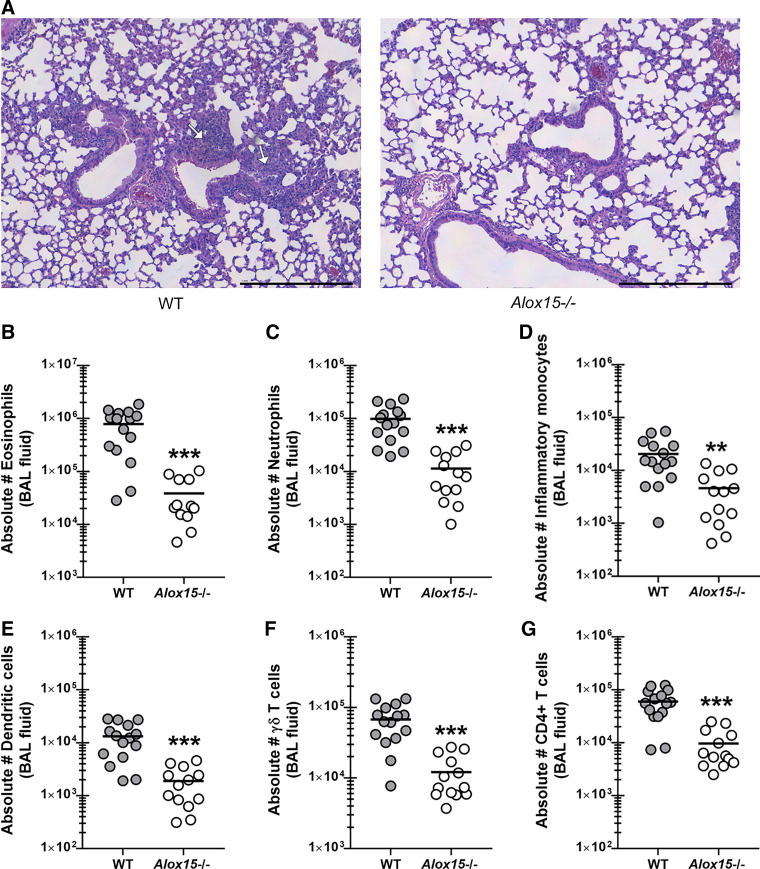
12/15-lipoxygenase (LOX) promotes increased lung cellularity during allergic fungal asthma. *A*: C57BL/6 wild-type (WT) and 12/15-LOX-deficient (*Alox15*^−/−^) mice were subjected to the experimental allergic fungal asthma model as described in materials and methods. Representative hematoxylin and eosin (H&E)-stained lung sections from asthmatic WT (*left*) and *Alox15*^−/−^ (*right*) mice. Original magnification ×10. Bar = 1 mm. White arrows represent areas of inflammation around the airways. Twenty-four hours after last challenge, lung cells from WT and *Alox15*^−/−^ mice were isolated by bronchoalveolar lavage, enumerated, Fc-blocked, stained with a live/dead staining kit, and then stained using cell surface markers for eosinophils (*B*), neutrophils (*C*), inflammatory monocytes (*D*), dendritic cells (*E*), γδ T cells (*F*), and CD4 T cells (*G*) and quantified via flow cytometry. The figure illustrates cumulative data from three independent studies (*n* = 4 or 5 mice per group, per study). Each dot presents a single mouse. The middle line represents the mean. Data are expressed as absolute cell number in lavage (BAL) fluid. **P < 0.01 and ***P < 0.001 (two-tailed Student’s *t* test).

We have previously reported that lower AHR is often correlated with lower lung inflammation and lung cellularity, usually associated with lower levels of neutrophils and eosinophils (17, 18). Histological analysis of lung sections from WT and *Alox15*^−/−^ mice revealed a decrease in inflammatory cells surrounding the blood vessels and the airways as well as infiltration into the alveolar spaces in *Alox15*^−/−^ mice (Fig. 3*A*, *right*) over that observed in WT mice (Fig. 3*A*, *left*). Quantifying these differences by flow cytometric analysis of cells isolated via bronchoalveolar lavage demonstrated that *Alox15*^−/−^ mice had significantly lower levels of eosinophils (Fig. 3*B*), neutrophils (Fig. 3*C*), inflammatory monocytes (Fig. 3*D*), dendritic cells (Fig. 3*E*), γδ T cells (Fig. 3*F*), and CD4 T cells (Fig. 3*G*). Thus, lower AHR in *Alox15*^−/−^ mice correlated with decreased levels of specific myeloid and lymphoid cells with recognized roles in asthma severity.

### 12/15-Lipoxygenase Promotes Type 1 and 2 and Multiple Chemotactic Responses during Allergic Fungal Asthma

**Figure 4.**
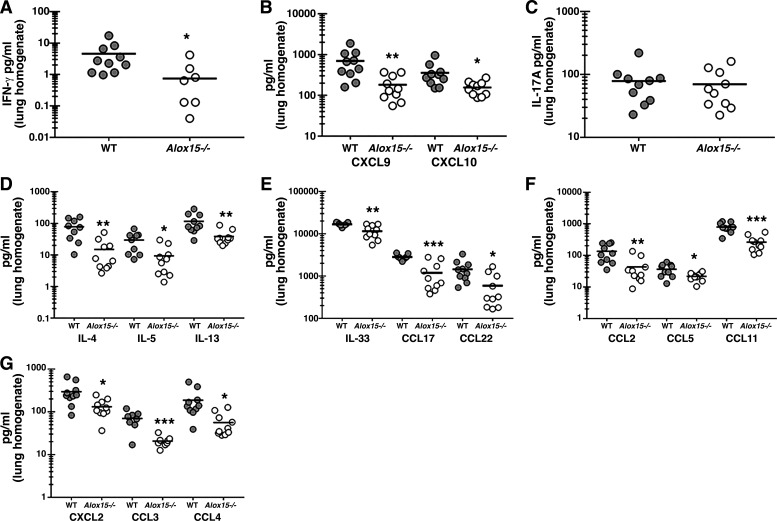
12/15-lipoxygenase (LOX) promotes type 1 and 2 and multiple chemotactic responses during allergic fungal asthma. *A–G*: C57BL/6 wild-type (WT) and 12/15-LOX-deficient (*Alox15*^−/−^) mice were subjected to the experimental allergic fungal asthma model as described in materials and methods. Twenty-four hours after last challenge, lungs were collected, homogenized, and clarified by centrifugation. IFN-γ (*A*), CXCL9 and CXCL10 (*B*), IL-17A (*C*), and IL-4, IL-5, and IL-13 (*D*) levels were quantified by Milliplex. IL-33, CCL17, and CCL22 (*E*) levels were quantified by ELISA. CCL2, CCL5, and CCL11 (*F*) and CXCL2, CCL3, and CCL4 (*G*) were quantified by Milliplex. The figures illustrate cumulative data from two independent studies (*n* = 5 mice per group, per study; note there are three zero values on the IFN-γ graph that are not visualized in log scale). Each dot presents a single mouse. The middle line represents the mean. Data are expressed as pg/mL in lung homogenate. *P < 0.05, **P < 0.01, and ***P < 0.001 (two-tailed Student’s *t* test).

Our previous work has shown that type 1/Th1 and type 17/Th17 responses often correlate with and contribute to disease severity during experimental fungal asthma (16–18). However, type 2/Th2 responses are also robustly induced during experimental fungal asthma and have been shown by others to also contribute to severity (26, 27). As there were specific changes in lung cellularity during experimental fungal asthma in *Alox15*^−/−^ mice, i.e., decreased myeloid and lymphoid populations, we questioned whether these changes correlated with changes in various type 1, type 2, type 17, and associated chemotactic responses. Results show that the type 1 cytokine IFN-γ (Fig. 4*A*) and the type 1-associated chemokines CXCL9 and CXCL10 (Fig. 4*B*) were significantly lower in asthmatic *Alox15*^−/−^ mice. However, the levels of IL-17A were not different between asthmatic WT and *Alox15*^−/−^ mice (Fig. 4*C*). In contrast, the type 2 cytokines IL-4, IL-5, and IL-13 were significantly lower in asthmatic *Alox15*^−/−^ mice (Fig. 4*D*) as were the pro-type 2 cytokine IL-33 and the type 2-associated chemokines CCL17 and CCL22 (Fig. 4*E*). Examination of chemokines associated with inflammatory monocyte and eosinophil recruitment, CCL2 and CCL11, respectively, were also observed to be significantly lower in asthmatic *Alox15*^−/−^ mice (Fig. 4*F*). CCL5, which may enhance activation of CD4 Th2 cells (28), was also significantly lower in asthmatic *Alox15*^−/−^ mice (Fig. 4*F*). Likewise, chemokines associated with neutrophil recruitment to the lungs, CXCL2, CCL3, and CCL4, were also significantly lower in asthmatic *Alox15*^−/−^ mice (Fig. 4*G*). Thus, 12/15-LOX functions as a negative regulator of type 1 and 2 responses during allergic fungal asthma.

### 12/15-Lipoxygenase Expression in Hematopoietic Cells Is Sufficient for Driving AHR during Allergic Fungal Asthma

**Figure 5.**
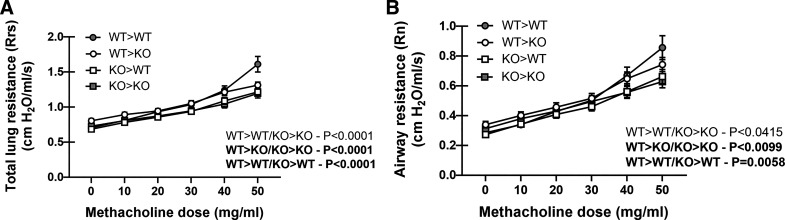
12/15-lipoxygenase expression in hematopoietic cells is sufficient for driving AHR during allergic fungal asthma. *A* and *B*: bone marrow chimeras were generated in which mice expressed 12/15-LOX in only the hematopoietic compartment (WT-KO), only the nonhematopoietic compartment (KO-WT), both compartments (WT-WT), or neither compartment (KO-KO) as described in materials and methods. Eight weeks following reconstitution, chimeras were chronically exposed to *Aspergillus fumigatus* as described in materials and methods. Total resistance (*A*) and airway (Newtonian) resistance (*B*) were measured via mechanical ventilation using the flexiVent pulmonary function system. The figure illustrates cumulative data from three independent studies (*n* = 4 or 5 mice per group, per study). Data are expressed as means ± SE. *P* values are included in the graph (two-way ANOVA). AHR, airway hyperresponsiveness.

12/15-LOX is most prominently expressed in monocytes and macrophages (29) although expression in endothelial cells has also been reported (30). To determine whether hematopoietic (myeloid) or nonhematopoietic 12/15-LOX expression contributed to allergic asthma severity, we created reciprocal bone marrow chimeras in which either the hematopoietic or the nonhematopoietic compartment expressed 12/15-LOX, as well as control chimeras in which both compartments (analogous to WT mice) or neither compartment (analogous to *Alox15*^−/−^ mice) expressed 12/15-LOX. Following reconstitution, these chimeras were subjected to experimental allergic fungal asthma, and airway function was assessed. This analysis revealed that expression of 12/15-LOX solely in the hematopoietic compartment (WT > KO) was sufficient for maintaining total lung resistance (Fig. 5*A*) and airway resistance (AHR) (Fig. 5*B*) compared with 12/15-LOX expressed solely in the nonhematopoietic (KO > WT) or in neither (KO > KO) compartment. This is reinforced when comparing lung function in the WT-WT group (12/15-LOX expressed in hematopoietic and nonhematopoietic cells) versus KO-WT chimeras (12/15-LOX expressed in nonhematopoietic cells only), i.e., the loss of 12/15-LOX expression in hematopoietic cells results in significantly lower AHR. We also compared AHR in the WT-WT group (12/15-LOX expressed in hematopoietic and nonhematopoietic cells) versus WT-KO chimeras (12/15-LOX expressed in hematopoietic cells only), which was not significantly different (*P* value = 0.8639, two-way ANOVA), i.e., the loss of 12/15-LOX in nonhematopoietic cells has no effect on AHR during allergic fungal asthma. Thus, hematopoietic/myeloid-expressed 12/15-LOX promotes responses that negatively affect lung function during allergic fungal asthma.

## DISCUSSION

The major lipoxygenases in humans include 5-LOX, 12-LOX, and 15-LOX, although leukocyte 12-LOX and reticulocyte 15-LOX form similar products and thus are usually referred to as 12/15-LOX (31). Mice express 5-LOX and 12/15-LOX, the latter encoded by the *Alox15* gene and is considered orthologous to human 12/15-LOX. Of these, 5-LOX is the most well-studied in asthma. 5-LOX drives the synthesis of the leukotrienes LTB4 and its cysteinyl derivatives LTC4 and LTD4, which are the main LTs identified in airways of patients with asthma (32, 33). In fact, cysteinyl leukotriene receptors are a thousand times more potent than histamine, making them an attractive drug target. Indeed, the novel 5-LOX inhibitor commercially known as Zileuton (34) and the cysteinyl leukotriene receptor antagonist commercially known as Montelukast (35, 36) are approved by the FDA as long-term asthma control medications. The role of 15-LOX in human asthma is increasing in appreciation, with data indicating that 15-LOX is induced by type 2 cytokines and is a prominent feature in type 2 eosinophilic asthma as well as aspirin-associated asthma (37, 38). Recent studies have implicated 15-LOX in glutathione redox changes in the asthmatic airway epithelium to worsen type 2 inflammation (39). Regarding 12/15-LOX, *Alox15*^−/−^ mice subjected to the OVA asthma model have generally demonstrated less severe asthma as measured by lung cellularity (22, 23). In the latter study, a protective effect of 12/15-LOX deficiency was only associated with local (lung) but not systemic sensitization (23). However, neither of these studies measured lung function as an outcome of asthma severity. An additional study also employed the OVA asthma model and demonstrated that 12/15-LOX overexpression leads to bronchial epithelial injury (24). The aeroallergen house dust mite (HDM) has been shown to be a potent inducer of 12/15-LOX-driven mediators (40), although the role of 12/15-LOX in HDM-driven asthma severity has not been determined. In contrast to the primary pathogenic role of 12/15-LOX in asthma, IL-33 administration to *Alox15*^−/−^ mice results in augmented airway inflammation, suggesting that 12/15-LOX expression is required to limit IL-33-mediated airway inflammation. Here again, however, lung function was not measured. Collectively, a clear role for 12/15-LOX has been difficult to ascertain, with studies indicating both protective and pathogenic roles, yet with no studies examining its role in lung physiological responses during asthma.

Our laboratory focuses on immunopathogenic versus immunoprotective mechanisms during fungal-associated allergic airway inflammation (fungal asthma). Regarding the latter, we have identified CX3CL1/fractalkine as an immunoprotective mediator during fungal asthma (41) as well as the chitinase-like protein BRP-39 (YKL-40 in humans), although BRP-39 protected the lung during fungal asthma in a yet to be defined nonimmune manner (19). In contrast, we have identified IL-7 (18) and IL-1R signaling (17) as immunopathogenic factors during fungal asthma. We have further demonstrated a role for IL-1RA in protecting the lung during fungal asthma and that IL-1RA (Kineret/anakinra) was effective immunoprotective therapeutic for improving fungal asthma severity (17). In the current report, we sought to elucidate the role of 12/15-LOX in an experimental animal model of fungal-associated allergic airway inflammation (fungal asthma). In humans, 12-LOX- and 15-LOX-mediated products, such as 15(S)-HETE (37), 5,15-diHETE, 13-HODE (40), and 12-HETE (24), are often detected in the asthmatic lung. In turn, our initial studies indicated that mice exposed to experimental fungal asthma had a fourfold increase in lung *Alox15* mRNA levels, which paralleled a threefold increase in the levels of the 12/15-LOX metabolite 12-HETE. In experimental animal models, assessment of total lung (dynamic) resistance, which is the level of constriction in the lungs, and Newtonian resistance, which is the level of constriction in the central and conducting airways, are hallmarks for evaluating the severity of asthma/allergic airway inflammation. Our findings indicate that products of 12/15-LOX function in some capacity to increase the level of constriction, as asthmatic mice deficient in 12/15-LOX demonstrated significantly better lung function compared with asthmatic WT control mice. In fact, lung function in asthmatic 12/15-LOX-deficient mice was near that of naïve 12/15-LOX-deficient mice, indicating the dramatic effect 12/15-LOX signaling has on the severity of allergic fungal asthma. This is the first report to our knowledge that examined the impact of 12/15-LOX on lung function during experimental allergic asthma.

Previous studies using the OVA model reported attenuated allergic airway inflammation in the absence of 12/15-LOX, specifically demonstrating lower IL-4, IL-5, and IL-13 protein levels in lung lavage fluid (22) and lower *Il4* and *Ifng*, but not *Il13*, mRNA levels in whole lung (23). Our study had partially similar findings, in that lung homogenate protein levels of IL-4, IL-5, and IL-13, as well as IFN-γ, were lower in mice deficient in 12/15-LOX. 12/15-LOX has been shown to be reciprocally regulated by IL-13 (increases) and IFN-γ (decreases) (42), suggesting that 12/15-LOX functions as an immunopathogenic factor in type 2/Th2 high asthma. Indeed, our data further demonstrated lower levels of CCL17 and CCL22, both of which have a well-described association with type 2 responses (43, 44). We further showed that the pro-type 2 cytokine IL-33, which has a pathogenic role in fungal asthma (27), was lower in asthmatic 12/15-LOX-deficient mice and correlated with the levels of type 2 mediators. Surprisingly, however, a previous report demonstrated that chronic administration of IL-33 drives higher type 2/Th2 responses in the absence of 12/15-LOX (25). This result suggests that 12/15-LOX is a negative regulator of type 2/Th2 responses, possibly when IL-33 levels are elevated. We have reported that type 1/Th1 and type 17/Th17 responses correlate with neutrophil levels and the severity of fungal asthma (16–18). Lower type 1/Th1 or type 17/Th17 responses could further explain better lung function in the absence of 12/15-LOX; although 12/15-LOX deficiency correlated with lower type 1/Th1 mediators (IFN-γ, CXCL9, CXCL10), there was no effect on the level of IL-17A. Finally, the levels of eosinophils, neutrophils, dendritic cells, inflammatory monocytes, γδ T cells, and CD4 T cells were lower in asthmatic 12/15-LOX-deficient mice, which correlated with the levels of chemokines associated with their recruitment, i.e., CCL11 (eosinophils), CXCL2/CCL3/CCL4 (neutrophils), CCL2 (inflammatory monocytes), and CCL5 (CD4 T cells).

Immunostaining of the lung from mice subjected to the OVA asthma model indicated that myeloid cells were the primary expressers of 12/15-LOX (24). Flow cytometry further demonstrated that eosinophils (Siglec F+), macrophages (CD11b+, F4/80+), dendritic cells (CD 11c+), T lymphocytes (CD3+), and granulocytes (Gr-1+) from asthmatic (OVA) mice had increased 12/15-LOX staining (24). Airway epithelial cells surprisingly did not express 12/15-LOX in asthmatic (OVA) mice, although epithelial cells from normal and human subjects with asthma had robust expression of 15-LOX (24). To further pinpoint in what cell(s) 12/15-LOX expression was required for driving worse lung function during experimental fungal asthma, we used reciprocal bone marrow chimeras to discriminate between the function of 12/15-LOX in hematopoietic cells (leukocytes) versus nonhematopoietic cells (lung epithelial cells, airway smooth muscle cells, etc.). We found that chimeras expressing 12/15-LOX in the hematopoietic compartment exhibited worse lung function compared with chimeras expressing 12/15-LOX only in the nonimmune compartment or in chimeras not expressing 12/15-LOX in either compartment. Comparisons between different chimeric groups further indicated that the loss of 12/15-LOX expression specifically in hematopoietic, but not nonhematopoietic, cells results in significantly lower AHR. From our flow cytometry data, we can hypothesize that 12/15-LOX expression in eosinophils, inflammatory monocytes, dendritic cells, and/or neutrophils promotes allergic fungal asthma severity. This is in line with a previous report showing that IL-13 stimulation of macrophages induced the 12/15-LOX metabolite 13-S-HODE, which promoted airway epithelial injury, as measured by reduced mitochondrial membrane potential and increased release of cytochrome C in cytosol (24). Ongoing studies are examining lipidomic differences between asthmatic WT and *Alox15*^−/−^ mice to pinpoint bioactive lipids that may serve as future mechanistic or therapeutic targets.

In conclusion, we report that 12/15-LOX is an immunopathogenic mediator in an experimental model of *Aspergillus*-associated allergic airway inflammation. Specifically, 12/15-LOX promoted type 1 and 2 responses, inflammatory cell recruitment, and worse AHR. 12/15-LOX expression in hematopoietic/myeloid cells promoted AHR, implicating immune/inflammatory cells as a source of 12/15-LOX metabolites that likely target airway epithelial or smooth muscle cells. It would be of interest to examine the role of myeloid-specific 12/15-LOX deficiency in an effort to further identify the myeloid cell subsets(s) that employs 12/15-LOX to promote immunopathogenesis during fungal asthma. Likewise, inhibiting 12/15-LOX or its metabolites, 15(S)-HETE (37), 5,15-diHETE, 13-HODE (40), or 12-HETE (24), could be attractive therapeutic targets for allergic asthma. Indeed, small molecule inhibitors for multiple (5, 12, and 15) lipoxygenases are being investigated for the treatment of a variety of diseases, including leukemia, heart disease, osteoarthritis, and heparin-induced thrombocytopenia. The findings presented here identify 12/15-LOX as a putative therapeutic target for the treatment of allergic fungal asthma.

## DATA AVAILABILITY

Data will be made available upon reasonable request.

## GRANTS

This work was supported by National Institutes of Health Grants HL122426 and HL136211 (both to C.S.).

## DISCLOSURES

No conflicts of interest, financial or otherwise, are declared by the authors.

## AUTHOR CONTRIBUTIONS

C.S. conceived and designed research; M.M., D.A.E., and M.J. performed experiments; M.M., D.A.E., M.J., and C.S. analyzed data; M.M. and C.S. interpreted results of experiments; M.M. and C.S. prepared figures; M.M. and C.S. drafted manuscript; M.M. and C.S. edited and revised manuscript; M.M., D.A.E., M.J., and C.S. approved final version of manuscript.
